# The novel BPRST classification for hemorrhoidal disease: A cohort study and an algorithm for treatment

**DOI:** 10.1016/j.amsu.2020.12.019

**Published:** 2020-12-19

**Authors:** Carlos Walter Sobrado, Carlos de Almeida Obregon, Lucas Faraco Sobrado, Lucas Morales Bassi, José Américo Bacchi Hora, Afonso Henrique Silva e Sousa Júnior, Sergio Carlos Nahas, Ivan Cecconello

**Affiliations:** Colorectal Surgery Division, Department of Gastroenterology, University of São Paulo School of Medicine, São Paulo, Brazil

**Keywords:** Hemorrhoids, Classification, Hemorrhoidectomy, Management

## Abstract

**Background:**

The classification for HD was developed by Goligher in 1980 and does not contemplate important aspects of this disease, which limits its use in guiding treatment. The aim of this study if to apply in clinical practice the new classification for hemorrhoids named BPRST (bleeding, prolapse, reduction, skin tags, thrombosis), to compare it with the original classification proposed by Goligher and to propose an algorithm for treatment.

**Materials and methods:**

This is a prospective study conducted at the University of São Paulo's teaching hospital and *Hospital 9 de Julho*. Patients with HD treated from March 2011 to July 2013 were included. Patients were classified according to BPRST and Goligher classifications and treated according to personal experience and most updated guidelines. The association between both classifications and the treatment adopted was compared and an algorithm for treatment was developed.

**Results:**

229 patients were included in this study and 28 patients were lost due to follow-up. According to Goligher, 29, 61, 85 and 26 were classified as grades I, II, III and IV, respectively. According to the BPRST, 23 were classified as stage I, 95 as stage II and 83 as stage III. Six patients classified as Goligher I were reclassified as BPRST stage III and required conventional hemorrhoidectomy, either due to thrombosis (n = 4) or intolerable skin tags (n = 2). The BPRST classification was more closely associated with the type of treatment employed and had few outliers than Goligher (p < 0.001).

**Conclusion:**

There are limitations to the use of Goligher's classification in clinical practice. The novel BPRST classification includes important aspects of HD that should be considered when deciding the best treatment option. Our algorithm for treatment contemplates the most commonly used techniques and can help to guide the treatment of this complex disease.

## Highlights

• A new classification for hemorroidal disease
• An algorithm for treatment of hemorrhoidal disease
• A cohort study of hemorrhoidal disease and a novel classification

## 1 Introduction

Hemorrhoidal disease (HD) is an important health issue due to its high prevalence and the impact on quality of life [1]. It is a complex disease with a myriad of clinical presentations and symptoms that can interfere with the choice of treatment, such as the association of external hemorrhoids, skin tags and acute thrombosis. However, to date, the most used classification in clinical practice was described in 1980 by Goligher, in which patients are classified in four grades based solely on the degree of prolapse and its reducibility. The algorithm that follows suggests that patients should be offered either clinical treatment for grades I and II or hemorrhoidectomy for grades III and IV [2].

We consider that there is a limitation to the use of this classification in clinical practice, which has also been suggested by other authors [[3], [4], [5]]**.** We have previously published a proposal for a new classification for hemorrhoids named BPRST, an acronym in which each letter that corresponds to an important aspect of HD that interferes with clinical reasoning: bleeding, prolapse, reducibility, skin tags and thrombosis. This classification was compared to Goligher's is a retrospective study at our institution, revealing good correlation with the treatment adopted but fewer discrepancies [6].

The aim of the present study is to analyze in a prospective study the BPRST classification in clinical practice, to compare with Goligher's classification and to propose an algorithm for treatment based on this new classification, the most updated guidelines and personal experience.

## 2 Methods

This is a prospective study conducted in two institutions. Patients were enrolled in this study if they were diagnosed with hemorrhoidal disease (HD) and were treated at the University of São Paulo Gastroenterology Department and Hospital 9 de Julho during the period from March 2011 to July 2013. This study was approved by the Institutional Review Board of University of São Paulo (Plataforma Brasil, CAAE 40778820.3.0000.0068) and informed consent was obtained before enrollment [7].

Patients were classified according to Goligher's (Table 1) and BPRST classifications (Table 2). Treatment was offered according to the most recent guidelines and personal experience at the discretion of the surgeon. The treatment options according to BPRST classification are shown in Table 3. This manuscript has been reported in line with the STROCCS criteria [8].

**Table 1:** Goligher's classification.

Grade	Degree of Prolapse
I	No prolapse
II	Prolapse on defecation with spontaneous reduction
III	Prolapse on defecation requiring manual reduction
IV	Irreducible prolapse

**Table 2:** BPRST classification: patients with bleeding only (B1) are classified as stage I (in red), patients with either P1, P2 or R1 are classified as stage II (in blue) and patients with R2, S1 or T1 are stage III (in green).

Bleeding (B)	Prolapse (P)	Reduction (R)	Skin Tag (S)	Thrombosis (T)
**B0** No bleeding	**P0** No prolapse	**R0** Spontaneous reduction	**S0** No skin tags	**T0** No thrombosis
**B1** Bleeding	**P1** Prolapse of 1 pile	**R1** Manual reduction	**S1** Symptomatic skin tags	**T1** With acute thrombosis
–	**P2** Prolapse of 2 or more piles	**R2** Irreducible prolapse	–	–

**Table 3:** HD treatment options according to BPRST Classification.

Hemorrhoidal Disease Staging and Therapeutic Options						
	Stage I	Stage II		Stage III		
	B_1_P_0_R_X_S_0_T_0_	B_1_P_1_R_0_S_0_T_0_	B_1_P_1/2_R_1_S_0_T_0_	Any R_2_	Any S_1_	Any T_1_
Lifestyle Changes	**✓**	**✓**	**✓**	**✓**	**✓**	**✓**
Sclerotherapy	**✓**	**✓**				
Rubber Band Ligation	**✓**	**✓**	**✓**			
Infrared Photocoagulation	**✓**	**✓**	**✓**			
Dearterialization with Mucopexy		a	**✓**	**✓**		
Stapled Anopexy		a	**✓**	**✓**		
Radiofrequency Ablation		a	**✓**	**✓**		
Hemorrhoidectomy				**✓**	**✓**	**✓**
Excision of Skin Tag					**✓**	
Thrombectomy						**✓**

a If failure of non-operative treatment.

Patients with bleeding HD without prolapse or external components, stage I BPRST, the recommended management involved medical therapy (dietary supplementation of fibers, behavioral measures, oral hydration, laxatives, ointments) and office procedures (rubber-band ligation, sclerotherapy or infrared photocoagulation) [1,6,[9], [10], [11], [12]].

For patients with prolapsed hemorrhoids that were reducible either spontaneously or manually (P1, P2 or R1), stage II BPRST, were recommended for non-anodermal excision surgical procedures, such as stapled anopexy (or procedure for prolapse and hemorrhoids - PPH), hemorrhoidal transanal dearterialization with mucopexy (THD) or radiofrequency ablation [1,[13], [14], [15]]**.**

For irreducible prolapse (R2), intolerable external components (S1) or with refractory acute hemorrhoidal thrombosis (T1), stage III BPRST, conventional hemorrhoidectomy with anodermal excision (Milligan-Morgan, Ferguson-Heaton, Obando, among other techniques) was the procedure of choice [1,6,[9], [10], [11], [12]]**.**

Statistical analysis was performed to examine the association between BPRST classification and the treatments that were performed. Fisher's exact test was used for the statistical analysis, in conjunction with confidence intervals (95% CI). The database was analyzed with the IBM Statistical Package for the Social Sciences (SPSS) 25.

## 3 Results

During the study period, a total of 229 patients were enrolled, 28 patients were lost to follow-up, resulting in 201 patients. Patient's profile and demographics are summarized in Table 4.

**Table 4:** Patient's profile and demographics.

Total of patients (n)	201
**Gender (male/female)**	130/71
**Age years (mean ± SD)**	48 ± 12
Goligher classification (n)	
Grade I	29
Grade II	61
Grade III	85
Grade IV	26
BPRST classification (n)	
Stage I	23
Stage II	95
Stage III	83

Under Goligher's classification, 29, 61, 95 and 26 were classified as grades, I, II, III and IV, respectively. Under the BPRST classification, 23, 95 and 83 were classified as stages I, II and III respectively.

**Fig. 1:**
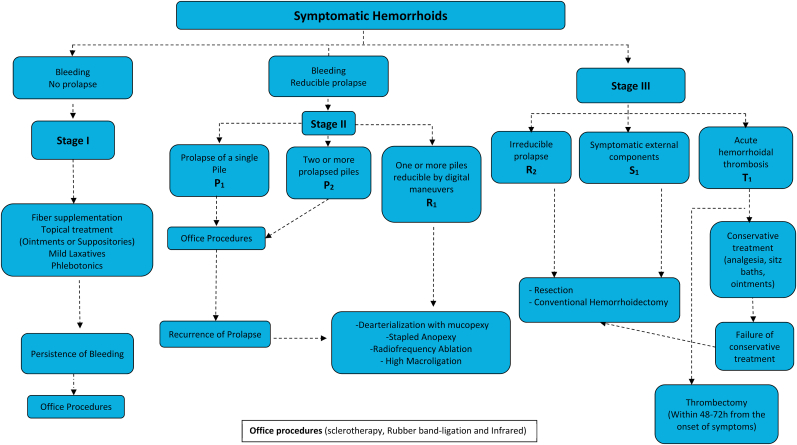
Proposed algorithm for treatment based on the novel BPRST classification.

Table 5 correlates Goligher's and BPRST classifications (p < 0.001). Out of 29 patients classified as Goligher grade I, therefore initially eligible for clinical treatment exclusively, 6 were reclassified as stage III BPRST and were treated with conventional hemorrhoidectomy. Patients with Goligher II, III were also reclassified as BPRST stages II and III. All patients with grade IV Goligher were classified as stage III BPRST. Table 6 details the differences in management for each group of patients. The proposed algorithm for treatment according to BPRST classification is shown in Fig. 1.

**Table 5:** Correlation between Goligher's and BPRST classifications^a^.

	BPRST Stage I	BPRST stage II	BPRST stage III
Goligher I	23 (79.3%)	0	6 (20.7%)
Goligher II	0	38 (62.3%)	23 (37.7%)
Goligher III	0	57 (67.1%)	28 (22.9%)
Goligher IV	0	0	26 (100%)

a p < 0.001.

**Table 6:** Correlation between Goligher's and BPRST managements*.

Goligher Classification	Techniques	N	%	N	%	N	%
				BPRST Classification			
		1		2		3	
1	Medical Treatment	19	82.6	0	0	0	0
	Rubber Band Ligation or Schlerotherapy	3	13	0	0	0	0
	Non-anodermal Excision Techniques	1	4.3	0	0	0	0
	Conventional Hemorrhoidectomy	0	0	0	0	6	100
		
2	Medical Treatment	0	0	8	21.1	1	4.3
	Rubber Band Ligation or Schlerotherapy	0	0	16	42.1	0	0
	Non-anodermal Excision Techniques	0	0	14	36.8	0	0
	Conventional Hemorrhoidectomy	0	0	0	0	22	95.7
							
3	Medical Treatment	0	0	2	3.5	0	0
	Rubber Band Ligation or Schlerotherapy	0	0	0	0	0	0
	Non-anodermal Excision Techniques	0	0	55	96.5	6	21.4
	Conventional Hemorrhoidectomy	0	0	0	0	22	78.6

*p < 0.001.

## 4 Discussion

Hemorrhoidal disease (HD) management is not straightforward due to the several clinical presentations and treatment options that are currently available. It is unreasonable to consider that one treatment could apply to all different presentations of this complex disease.

The current used classification in clinical practice was proposed by Goligher in 1980 and considers solely the internal aspect of the disease: the prolapse and its reducibility. It suggests that grades I and II should be offered clinical treatment whereas grades III and IV should undergo conventional hemorrhoidectomy.

While this was certainly true in 1980 when the classification was developed, at the 21-century many new techniques have been developed and became part of the colorectal surgeon's arsenal, such as the Transanal Hemorrhoidal Dearterialization (THD) procedure, described by Morinaga in 1995 [14], and the Procedure for Prolapse and Hemorrhoids (PPH), described in 1998 by Longo [13], both of which have been widely used in clinical practice, including at our institution [16].

There is a general consensus among other experts in the field that some form of revision is needed for the classification of hemorrhoids [[3], [4], [5]], [10], [17], however, new classifications have failed to be used in clinical practice mainly due to its complexity and lack of practical use in guiding treatment. The novel classification BPRST intends to be holistic, to contemplate the different aspects of the disease and also the several forms of treatment that are currently available.

The algorithm we propose for treatment of HD using the BPRST classification was based on updated guidelines [1,10,18,19] and personal experience at a specialized center for colorectal disease at the University of São Paulo. It does not intend to be exclusive but rather to serve as a guide for clinicians and colorectal surgeons in the treatment of these patients. Other forms of treatment are accepted but should be analyzed on an individual basis.

It is interesting to note that despite a clear correlation between Goligher's and BPRST classification in our group of patients, the novel classification was more accurate in terms of staging the disease and guiding treatment. Out of 29 patients who were previously classified as Goligher grade I, therefore originally considered for clinical treatment, 6 (20.7%) had to undergo surgical treatment with conventional hemorrhoidectomy, either due to thrombosis (n = 4) or symptomatic skin tags (n = 2), conditions that are very frequent in clinical practice.

For patients classified as Goligher grade II, 36 (62.3%) we reclassified as BPRST stage II and 23 (37.7%) as stage III. For these patients, rubber band ligation was required in 16, non-excisional methods in 14, and conventional hemorrhoidectomy in 22. This illustrates that there are situations where Goligher I and II HD require surgical treatment, which are not contemplated by the original classification.

This is a single institution study aimed to compare this novel classification for hemorrhoids named BPRST with the one proposed by Goligher in 1980. The algorithm for treatment based on the BPRST classification still has to be validated in a multicenter study before it can be generally applied to clinical practice. However, we share the opinion that some form of revision in terms of classification of HD is needed and this may be the first step.

## 5 Conclusion

There are limitations to the use of Goligher's classification in clinical practice. The BPRST classification includes important aspects of hemorrhoid disease that should be considered when deciding the best treatment option. Our algorithm for treatment contemplates the most used techniques and can help to guide the treatment of this complex disease.

## Funding for your research

No funding to disclose.

## Ethical approval

This study was authorized by the Institutional Review Board of University of São Paulo (No. 05156818.2.0000.0068) and informed consent was obtained before enrollment.

## Consent

Written informed consent was obtained before enrollment.

## Author contribution

Sobrado CW: conceptualization, study design, writing original article and review.

Obregon CA, Sobrado LF and Bassi LM: writing original article, review and data acquisition.

Hora JAB and Sousa Junior AHS: review and literature search.

Cecconello I and Nahas SC: critical review and supervision.

## Registration of research studies

1 Name of the registry: CAAE
2 Unique Identifying number or registration ID: 40778820.3.0000.0068
3 Hyperlink to your specific registration (must be publicly accessible and will be checked) https://plataformabrasil.saude.gov.br/

## Guarantor

Carlos Walter Sobrado.

## Declaration of competing interest

No conflicts of interest to disclose.

## References

[1] Rivadeneira D.E., Steele S.R., Ternent C., Chalasani S., Buie W.D., Rafferty J.L. (2011 Sep). Standards practice task force of the American society of colon and rectal surgeons. Practice parameters for the management of hemorrhoids (revised 2010). Dis. Colon Rectum.

[2] Goligher J.C. (1980). Haemorrhoids or piles. Surgery of the Anus, Rectum and Colon.

[3] Elbetti C., Giani I., Novelli E., Fucini C., Martellucci J. (2015 Dec). The single pile classification: a new tool for the classification of haemorrhoidal disease and the comparison of treatment results. Updates Surg.

[4] Gerjy R., Lindhoff-Larson A., Nyström P.O. (2008 Sep). Grade of prolapse and symptoms of haemorrhoids are poorly correlated: result of a classification algorithm in 270 patients. Colorectal Dis..

[5] Rubbini M., Ascanelli S., Fabbian F. (2018 Jun). Hemorrhoidal disease: is it time for a new classification?. Int. J. Colorectal Dis..

[6] Sobrado C.W., Obregon C.A., Silva e Sousa Junior A.H., Sobrado L.F., Nahas S.C., Cecconello I. (2020 Aug). A new classification for hemorrhoidal disease: the creation of the "BPRST" staging and its application in clinical practice. Ann Coloproctol.

[7] Plataforma Brasil. https://plataformabrasil.saude.gov.br/.

[8] Agha RA, Abdall-Razak A, Crossley E, Dowlut N, Iosifidis C, Mathew G, for the STROCSS Group. STROCSS 2019 Guideline: Strengthening the Reporting of Cohort Studies in Surgery. 10.1016/j.ijsu.2019.11.002 31704426

[9] Sneider E.B., Maykel J.A. (2010). Diagnosis and management of symptomatic hemorrhoids. Surg. Clin..

[10] Rubbini M., Ascanelli S. (2019). Classification and guidelines of hemorrhoidal disease: present and future. World J. Gastrointest. Surg..

[11] Yamana T. (2018 May 25). Japanese practice guidelines for anal disorders I. Hemorrhoids. J Anus Rectum Colon.

[12] Reis Neto J.A., Quilici F.A., Cordeiro F., Reis Junior J.A. (1989). Tratamento ambulatorial de Hemorroidas: estudo prospectivo randomizado. Rev Bras Colo-Proct..

[13] Longo A. (1998). Treatment of haemorrhoids disease by reduction of mucosa and haemorrhoidal prolapse with a circular suturing device: a new procedure. Proceedings of the 6th World Congress of Endoscopic Surgery.

[14] Morinaga K., Hasuda K., Ikeda T. (1995 Apr). A novel therapy for internal hemorrhoids: ligation of the hemorrhoidal artery with a newly devised instrument (Moricorn) in conjunction with a Doppler flowmeter. Am. J. Gastroenterol..

[15] Gupta P.J. (2002). Novel technique: radiofrequency coagulation - a treatment alternative for early-stage hemorrhoids. MedGenMed: Medsc. Gen. Med..

[16] Sobrado C.W., Cotti G.C.C., Coelho F.F., Rocha J.R.M. (2006). Initial experience with stapled hemorrhoidopexy for treatment of hemorrhoids. Arq. Gastroenterol..

[17] Lunniss P.J., Mann C.V. (2004 Jul). Classification of internal haemorrhoids: a discussion paper. Colorectal Dis..

[18] Trompetto M., Clerico G., Cocorullo G.F., Giordano P., Marino F., Martellucci J. (2015 Oct). Evaluation and management of hemorrhoids: Italian society of colorectal surgery (SICCR) consensus statement. Tech. Coloproctol..

[19] Gallo G, Martellucci J, Sturiale A, Clerico G, Milito G, Marino F, Cocorullo G, Giordano P, Mistrangelo M, Trompetto M (2020 Feb). Consensus statement of the Italian society of colorectal surgery (SICCR): management and treatment of hemorrhoidal disease. Tech. Coloproctol..

